# Assessment of pulmonary arterial enhancement on CT pulmonary angiography using a leg vein for contrast media administration

**DOI:** 10.1097/MD.0000000000009099

**Published:** 2017-12-08

**Authors:** Cherry Kim, Choong Wook Lee, Gil-Sun Hong, Gihong Kim, Ki Yeol Lee, Sung-Soo Kim

**Affiliations:** a Department of Radiology and the Research Institute of Radiology, University of Ulsan College of Medicine, Asan Medical Center, Seoul; b Department of Radiology, Ansan Hospital, Korea University College of Medicine, Ansan-si, Gyeonggi; c Department of Healthcare Management, Cheongju University, Cheongju, South Korea.

**Keywords:** contrast media, CT pulmonary angiography, pulmonary embolism, vascular enhancement, venous route

## Abstract

The purpose of our study was to compare pulmonary artery (PA) enhancement according to venous routes of contrast media (CM) administration in patients who underwent CT pulmonary angiography (CTPA) in the emergency department (ED).

This retrospective study reviewed the CTPAs of 24 patients who administered CM via leg veins (group A) and 72 patients via arm veins (group B) with age and gender matching at a ratio of 1:3. Clinical data, aorta attenuation (Ao_atten_), and PA attenuation (PA_atten_) were compared between group A and B. Each group was subcategorized into diagnostic and nondiagnostic CTPA subgroups, with a threshold of 250 HU at the PA. Then, clinical data, rates of pulmonary embolism (PE), and right ventricle (RV) strain were compared. In group A, the relationship between the narrowest suprahepatic IVC area (IVC_area_) and the attenuation ratio of the RV to the intrahepatic IVC (RV/IVC_atten_) was evaluated.

Ao_atten_ (236.6 HU vs 293.1 HU, *P* *<* .001) and PA_atten_ (266.7 HU vs 321.4 HU, *P* *=* .026) were significantly lower in group A than in group B. The proportion of nondiagnostic CTPA was significantly higher in group A than in group B (58.3% vs 19.4%, *P* = .001). In the subgroup analysis in of group A, patients with a nondiagnostic CTPA were significantly younger (55.3 years vs 68.6 years, *P* *=* .026) and showed a significantly lower incidence rate of PE (14% vs 70%, *P* = .01) than patients with a diagnostic CTPA. However, the radiological diagnostic rate of RV strain was comparable between patients with nondiagnostic and diagnostic CTPA. In group A, IVC_area_ and RV/IVC_atten_ were positively correlated, with a correlation coefficient of 0.430 (*P* *<* .036).

In conclusion, administration of CM through the leg veins increases the nondiagnostic CTPA rate, reducing the detection rate of PE. When CM is injected via the leg veins, the degree of PA enhancement is related with to the diameter of the suprahepatic IVC; therefore, adjustment of respiratory maneuvers may be needed to promote IVC flow into the right cardiac chamber, and to improve PA enhancement.

## 1 Introduction

CT pulmonary angiography (CTPA) is the clinical standard for the detection of pulmonary embolism (PE).^[1–4]^ Optimization of image quality is essential for good diagnostic performance of CTPA. The degree of pulmonary artery (PA) enhancement is critical in detecting PE by CTPA. Because PE appears as a filling defect inside the contrast-enhanced PA on CTPA, a higher degree of PA enhancement increases the lesion contrast of PE. CTPA, however, may be inadequate for reasons such as poor PA enhancement, parenchymal disease, patient habitus, motion artifacts, and streak artifacts.^[5]^ Poor PA enhancement may be adjusted by utilizing proper methods of contrast media (CM) administration. CM protocols, such as optimal scan timing window, proper amount of CM, and selection of the best intravenous (IV) access route, are directly related to CTPA image quality.

The routine protocol for CTPA usually involves IV administration of CM through an access route in the arm. In the emergency department (ED), however, some patients have venous access only in the leg. When CM is administered through leg veins, the degree of PA enhancement is directly related to the amount of right atrial inflow from the inferior vena cava (IVC) as well as the contrast injection protocol. A retrospective study in pediatric patients showed that the degree of PA enhancement was similar when CM was administered through arm and leg veins.^[6]^ However, to our knowledge, the effects of venous routes on CTPA imaging quality has not been evaluated in adult patients.

This study was therefore designed to assess the degree of PA enhancement in adult patients in whom CM was administered through a leg vein during CTPA in the ED.

## 2 Materials and methods

### 2.1 Study population and clinical data

This retrospective study was approved by the institutional review board of our hospital. From January 2012 to January 2013, 1521 patients underwent CTPA for evaluation of PE in the ED. Among them, 24 patients (group A) received CM through a leg vein because an arm vein was inaccessible. Reasons for not administering CM into the arm vein included superior vena cava (SVC) syndrome (n = 3), splinting of both arms due to multiple traumas (n = 2), failure of several attempts to gain a venous access route in the arm (n = 5), and nonrecorded causes (n = 14). Patients in group A were matched for age and gender at a ratio of 1:3 with 72 consecutive control patients who underwent CTPA with venous access in an arm vein (group B). We excluded patients less than 18 years old, and those with exams that were not conducted using standard protocols. Veins in the anticubital fossa or dorsum of the hands were used for arm veins, and veins in the calves or the dorsum of the feet were used for leg access. Finally, a total of 96 patients were included in this study. The electronic medical records of all 96 patients in both groups, including their age, gender, body weight, and history of cardiac disease, were reviewed.

### 2.2 CT imaging technique

All CT scans were performed with a 128-channel MDCT scanner (SOMATOM definition plus, Siemens, Erlangen, Germany) with the subject in the supine position. All images were obtained in a caudocranial direction from the lung bases through the thoracic inlet level during a single inspiratory breath-hold. Except 2 patients who had an injury in both arms, all patients were examined with their arms elevated above their head. The CT scan parameters included tube voltage 120 kVp; tube current 200 mA; beam collimation 0.6 mm; rotation time 0.5 seconds; pitch 1.2; and reconstructed image thickness 1.5 mm. All examinations included multiplanar reconstructions in the coronal plane.

All patients were administered a bolus of 100 mL of nonionic CM (Iomeprol 400 mgI/mL, Iomeron, Bracco, Milan, Italy) at a rate of 3.5 mL/second through a 20-gauge cannula into an arm or leg vein using a power injector (Stellant, Medrad, Indianola, IN). Immediately after administration of CM, a 40-mL saline bolus was injected at the same rate. A bolus tracking technique triggered at 100 Hounsfield units (HU) on the pulmonary trunk was used with a delay of 10 seconds before scanning. Automated verbal breathing instructions were used during the scanning. Patients were instructed to take a deep breath in and hold it just before the scan started.

### 2.3 Image analysis

All images were stored on a picture archiving and communication system (PACS) workstation for evaluation, and the optimal window settings were adjusted for each subject. All images were retrospectively reviewed by 2 board-certified radiologists (C.K. and C.W.L.), with decisions reached by consensus. The vascular enhancement of the IVC at the intrahepatic level (group A) and SVC at the right pulmonary arterial level (group B) was evaluated to confirm that CM was present in the precardiac venous system.

Attenuation was measured by placing square-shaped regions of interest (ROIs) over the main pulmonary trunk, right and left PAs, aortic arch, and ascending and descending aorta. The size of each ROI was adjusted to be as large as possible, avoiding partial-volume and streak artifacts. After each anatomical area was measured, the mean PA attenuation (PA_atten_) was calculated as the average attenuation in the main pulmonary trunk and the right and left PAs. Mean aortic attenuation (Ao_atten_) was calculated by averaging the attenuation in the ascending aorta, aortic arch, and descending aorta. CTPA quality was assessed by the degree of mean PA_atten_, and dichotomized into 2 subgroups: diagnostic CTPA (mean PA_atten_ ≥ 250 HU) and nondiagnostic CTPA (mean PA_atten_ < 250 HU).^[5,7]^ Nondiagnostic CTPA was subcategorized as very poor (mean PA_atten_ < 100 HU), poor (100 ≤ mean PA_atten_ < 200 HU), or suboptimal (200 ≤ mean PA_atten_ < 250 HU) degrees. Additionally, in group A, the attenuation of the intrahepatic IVC and right ventricle (RV) were measured, and the area of the suprahepatic IVC at the narrowest point was also measured on axial CT image.

PE was diagnosed when intravascular filling defects were identified in at least one PA. RV strain was radiologically diagnosed when axial CT images showed both right ventricular enlargement and interventricular septal flattening (i.e., a D-shaped left ventricle).^[8,9]^

### 2.4 Statistical analysis

Clinical data, Ao_atten_, PA_atten_, and CTPA quality were compared between group A and B, using Fisher exact test, Student *t* test, or the Mann–Whitney *U* test, as appropriate. Between diagnostic and nondiagnostic CTPA subgroups of each group, clinical data (age and body weight) and rates of PE and RV strain were compared, using Fisher exact test or the Mann–Whitney *U* test. In group A, the relationship between the narrowest suprahepatic IVC area (IVC_area_) and the attenuation ratio of the RV to the intrahepatic IVC (RV/IVC_atten_) was evaluated using Pearson correlation test.

A *P*-value less than .05 was considered statistically significant. All statistical analyses were performed by statistical expert (S.K.) with a commercial software package (SPSS, version 19.0; SPSS, Chicago, IL).

## 3 Results

### 3.1 Clinical data and CT attenuation

Clinical data and CT attenuations are described in Table 1. There were no statistically significant differences in age, gender, body weight, and history of cardiac disease between groups A and B. Three patients in group A and 9 patients in group B had a history of cardiac disease (*P* *>* .999), including variant angina (n = 3) in group A and, acute myocardial infarction (n = 4), variant angina (n = 2), atrial fibrillation (n = 2), and mitral valve regurgitation (n = 1) in group B.

**Table 1:**
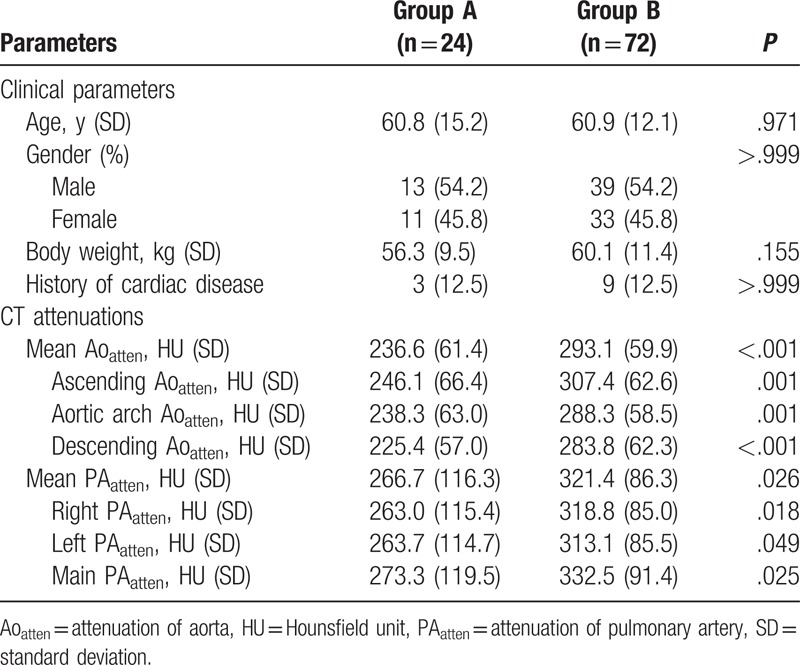
Comparison of clinical data and CT attenuations.

In all enrolled CTPAs, nonmixed high-attenuation CM was present in the precardiac venous system in the IVC (group A) and SVC (group B). The mean Ao_atten_ (236.6 HU vs 293.1 HU, *P* *<* .001) and mean PA_atten_ (266.7 HU vs 321.4 HU, *P* *=* .026) were significantly lower in group A than in group B, as were all measured attenuations in each segment of the aorta and PA (Table 1).

When mean PA_atten_ was dichotomized into 2 subgroups based on 250 HU, the CTPA was nondiagnostic (mean PA_atten_ < 250 HU) in 29.1% (28/96) of all enrolled patients, with a significantly higher rate in group A (58.3%, 14/24) than in group B (19.4%, 14/72) (*P* *=* .001) (Table 2). The subcategory distribution of nondiagnostic subgroups showed significant difference between group A and B (*P* *=* .046): 6/14 (42.9%) had a suboptimal mean PA_atten_ in group A, and 12/14 (85.7%) had a suboptimal mean PA_atten_ in group B.

**Table 2:**
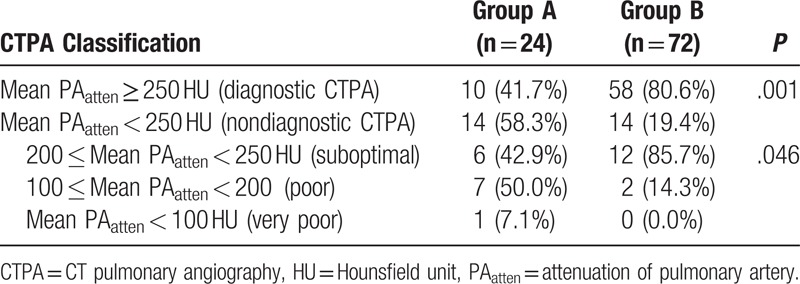
Comparison of CT pulmonary angiography classification according to the mean attenuation of pulmonary arteries.

Nine patients (37.5%) in group A and 22 patients (30.6%) in group B had PE (*P* *=* .616). RV strain was radiologically diagnosed in 7 patients (29.2%) in group A and 8 patients (11.1%) in group B (*P* *=* .051).

### 3.2 Subgroup comparison: diagnostic vs nondiagnostic subgroups

In group A, the nondiagnostic subgroup was significantly younger (55.3 years vs 68.6 years, *P* *=* .026) and showed a lower incidence rate of PE (14.3% vs 70.0%, *P* *=* .010) than the diagnostic subgroup, but the radiological diagnostic rate of RV strain was comparable (Table 3). In group B, the incidence rate of PE (28.6% vs 31.0%, *P* *>* .999) and the radiological diagnostic rate of RV strain were similar in the nondiagnostic and diagnostic subgroups, but body weight was greater in the nondiagnostic subgroup.

**Table 3:**
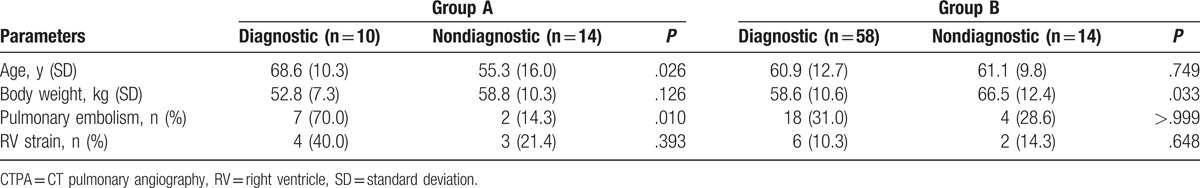
Comparison of clinical data and rates of pulmonary embolism and right ventricle strain between diagnostic CT pulmonary angiography (CTPA) and nondiagnostic CTPA in each group A and B.

### 3.3 Relationship between IVC_area_ and RV/IVC_atten_

**Figure 1:**
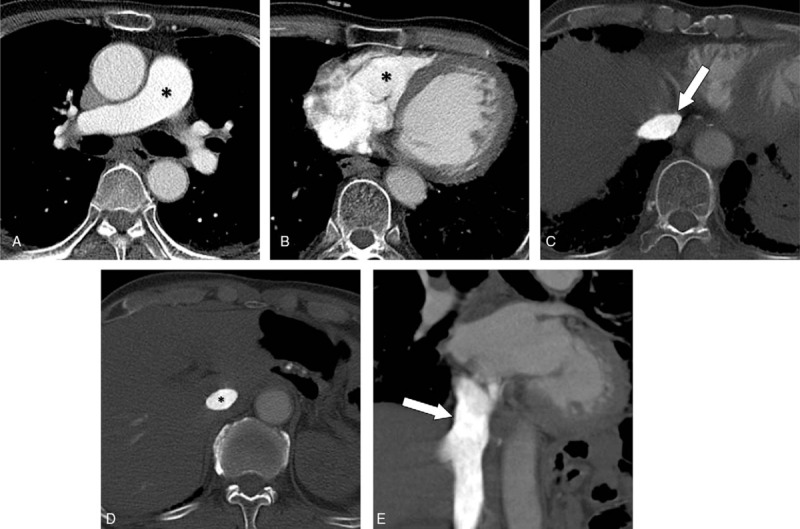
CT pulmonary angiography (CTPA) images from a 68-year-old man (group A) with a patent IVC and good enhancement in the pulmonary artery (PA). (A) PA attenuation was 311 HU, classified as a diagnostic CTPA (asterisk). (B and D) The attenuations of the RV and intrahepatic IVC were 376 and 823 HU (asterisks), respectively, with an IVC/RV attenuation ratio of 0.46. (C and E) The area of the suprahepatic IVC at the narrowest point was 300 mm^2^ (arrows).

**Figure 2:**
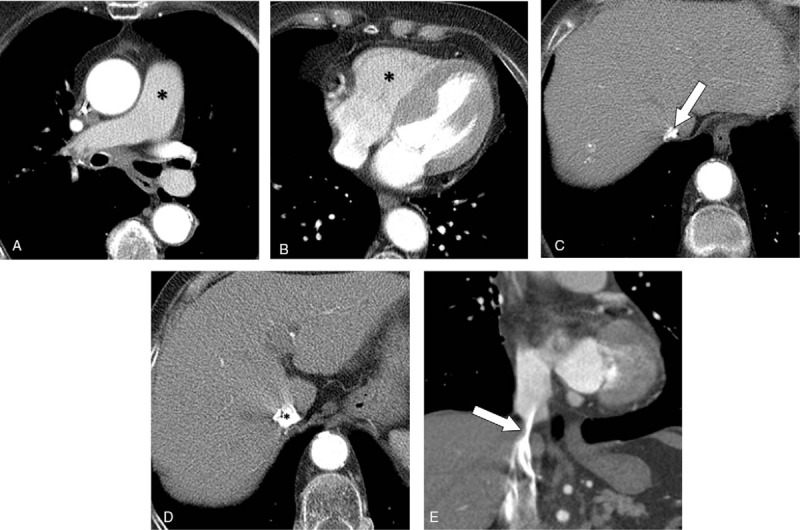
CT pulmonary angiography (CTPA) images from an 81-year-old woman (group A) with a stenotic IVC and poor enhancement in the pulmonary artery (PA). (A) PA attenuation was 178 HU, classified as a nondiagnostic CTPA (asterisk). (B and D) The attenuations of the RV and intrahepatic IVC were 167 and 736 HU (asterisks), respectively, with an IVC/RV attenuation ratio of 0.23. (C and E) The area of the suprahepatic IVC at the narrowest point was 61 mm^2^ (arrows).

**Figure 3:**
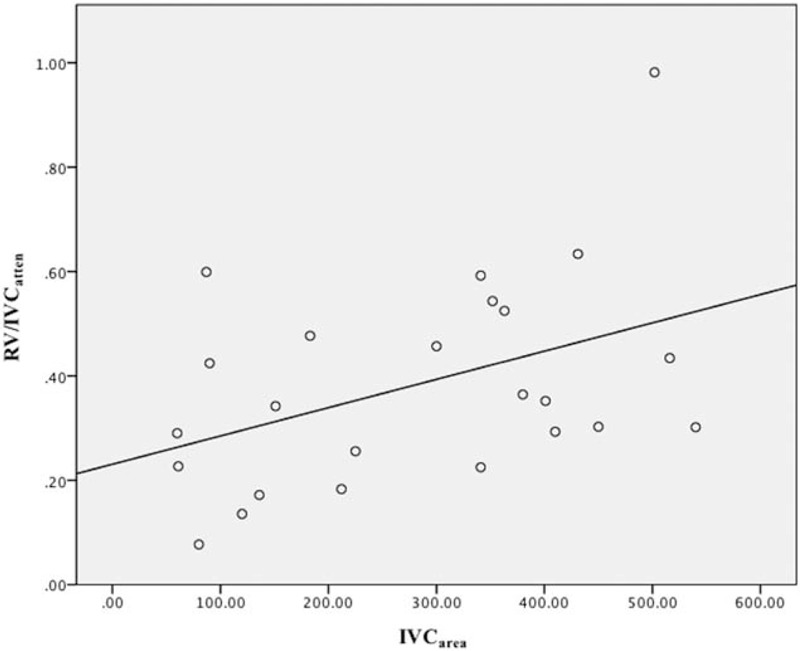
The scatter plot between the area of IVC (IVC_area_) and the attenuation ratio of RV to IVC (RV/IVC_atten_) in group A, shows positive relationship with a linear correlation coefficient of 0.430 (*P* < .036).

The representative cases of group A are shown in Figs. 1 and 2. The scatter plot between the IVC_area_ and the RV/IVC_atten_ in group A is presented in Fig. 3. The linear correlation coefficient was 0.430 (*P* = .036), indicating that the attenuation ratio of RV to IVC is positively correlated with the narrowest IVC area when CM is administered via the leg vein.

## 4 Discussion

This study demonstrated that nondiagnostic CTPAs were significantly more frequent (*P* = .001) and mean PA_atten_ was significantly lower (266.7 HU vs 321.4 HU, *P* = .026) when CM was injected into the leg vein compared to the arm vein. The rate of very poor (mean PA_atten_ < 100 HU) and poor quality (100 ≤ mean PA_atten_ < 200 HU) nondiagnostic CTPAs was significantly higher when CM was injected into the leg vein compared to the arm vein (*P* = .046). In addition, the RV/IVC_atten_ ratio showed a linear relationship with the narrowest IVC area when CM was injected into the leg vein.

PA_atten_ correlates directly with the degree of enhancement of the right cardiac chamber. Because blood from the SVC mixes with blood from the IVC in the right atrium, the mean attenuation of the right cardiac chamber is influenced by the CT attenuations of the SVC and IVC and their relative amount of right atrial filling. If CM is administered via an arm vein, the attenuation of the right cardiac chamber is determined by the ratio of the high-attenuation iodinated CM from the SVC and the low-attenuation blood from the IVC. A high SVC/IVC flow ratio would increase CT attenuation of the right cardiac chamber and, consequently, of the PA. The mixing ratio of SVC and IVC blood does not remain constant, but is affected primarily by the respiratory phase and the Valsalva maneuver. Several previous studies showed that increased blood flow from the IVC during inspiration interrupts main PA attenuation.^[10–12]^ A study using MRI to assess caval blood flow during respiratory maneuvers found that total blood flow from the IVC decreased during the breath-hold after inspiration, which is the same type of respiratory maneuver used in the routine CTPA protocol.^[11]^ In addition, total blood flow from the IVC was significantly decreased during a Valsalva maneuver (82 mL/heartbeat) or during the breath-hold at the end of inspiration (90 mL/heartbeat) compared to breathing state (110 mL/heartbeat, *P* < .01). That is, at the end of inspiration or during a Valsalva maneuver, the relative flow volume from the SVC increases, resulting in better PA enhancement when CM is administered via an arm vein; conversely, this results in poor PA enhancement when CM is administered via a leg vein. In addition, the linear relationship that we observed between the RV/IVC_atten_ and the narrowest IVC area in group A indicates that right cardiac blood inflow from the IVC is directly related to the narrowest IVC area. This suggests that diaphragmatic contraction during inspiration results in narrowing of the suprahepatic IVC, disturbing the cardiac blood inflow from the IVC. Therefore, relief of the diaphragmatic contraction and intraabdominal pressure may promote CM-containing blood inflow from the IVC and enhance PA effectively during the CT scan, when CM is administered via the leg vein.

In this study, patient age (in group A) and body weight (in group B) influenced the degree of PA enhancement. In patients receiving CM through the leg vein (group A), the nondiagnostic CTPA subgroup was significantly younger than the diagnostic CTPA subgroup (55.3 years vs 68.6 years, *P* *=* .026). This result may be explained by the age-related functional ability of breath holding and the Valsalva maneuver. As younger patients may be better able to follow directions about respiration during scanning (i.e., breath-holding at the end of inspiration), blood flow from the CM-containing high-attenuation IVC blood may be interrupted more in younger patients than in older patients. In patients receiving CM through the arm vein (group B), the body weight was significantly lower in patients with diagnostic CTPA. This finding was in agreement with previous studies, which reported that patient body size (weight and height) was one of the most important patient-related factors affecting contrast enhancement.^[7,13–16]^

Subgroup analysis of patients receiving CM through a leg vein showed that PE was significantly more frequent in patients with diagnostic than nondiagnostic CTPA (70.0% vs 14.3%, *P* = .010). However, the rate of RV strain, an ancillary finding of PE that is not influenced by the degree of PA enhancement, did not differ significantly in these 2 subgroups (40.0% vs 21.4%, *P* = .393). If the prevalence of PE is presumed to be the same in both the diagnostic and nondiagnostic subgroups based on the rate of RV strain, then the rate of false-negative results would be higher in the nondiagnostic CTPA subgroup. However, the prevalence of PE was similar in subgroups of patients with diagnostic and nondiagnostic CTPA receiving CM through an arm vein (31.0% vs 28.6%, *P* > .999). Of the nondiagnostic CTPAs in this group, 85.7% showed suboptimal quality, but none showed very poor quality, perhaps explaining why most PEs could be detected, even in nondiagnostic CTPAs of patients receiving CM through the arm vein.

Meanwhile, the PE detection rate seemed to be higher in group A diagnostic subgroup than in group B diagnostic subgroup (70% vs 31%). In group A, CM was administration via leg vein due to SVC syndrome (12.5%, 3/24) and severe trauma with bilateral arm injury (8.3%, 2/24), which both are accompanied by a very high risk for developing PE. We believe those patients with high risk for PE have skewed the PE incidence higher PE detection rate in group A, which may have led to the difference of the incidence of PE and associated RV strain between group A and group B (RV strain, 40% vs 10.3%).

We found that CTPA was nondiagnostic in 25.0% of patients, including in 58.3% of patients receiving CM through a leg vein and 19.4% of those receiving CM through an arm vein. Previous studies reported rates ranging from 6% in the general population to 25% in ICU patients.^[5,17,18]^ Although the rate of nondiagnostic CTPA in our study was higher than in the general population, it was acceptable, inasmuch as our study population consisted of only ER patients with acute illnesses. However, nondiagnostic CTPA was observed in 58.3% of patients receiving CM through the leg vein, which means that specific concerns about the respiration maneuver should be considered when CTPA is performed using a venous route in the leg.

This study had several limitations, the most important being its retrospective nature. Although all patients were instructed to follow the automated verbal breathing instructions, actual breathing motion was not checked during the examination. Therefore, variations in respiratory motion may have influenced the study results. Other factors, such as cardiac output and pulmonary function, which may have affected the dynamics of the CM, but these data were not available because few patients underwent cardiac imaging or pulmonary function tests at the time of CTPA. However, examination of the history of cardiac disease in these patients showed no significant differences between the 2 enrolled groups. This study also could not investigate imaging processing functions that could improve the image quality of CTPA. In addition, because more than half of the cause of leg injection were unknown, we could not statistically analyze whether these could potentially increase the incidence rate of nondiagnostic CTPA. The second limitation was the absence of other standard diagnostic methods to detect PE. Former standard methods, such as ventilation/perfusion scan and pulmonary angiography, are not currently used for the diagnosis of PE, because CTPA is regarded as the most sensitive method.^[2]^ Because the degree of pulmonary arterial enhancement is related to the detection, but not the actual presence, of PE, we assumed that the prevalence of PE was similar in both diagnostic and nondiagnostic CTPA subgroups. This was also verified indirectly by the similar rates of RV strain in the 2 subgroups. Another limitation was the arbitrary CT attenuation cut-off value (<250 HU) for defining diagnostic CTPA, which was based on previous results.^[5,7]^ However, another study used a CT attenuation cut-off of 200 HU.^[19]^ In our study, a CTPA in which the CT attenuation of the PA ranged from 200 to 250 HU was defined as of “suboptimal quality.” The third limitation is the fixed amount of IV contrast (100 mL) for every patient, instead of using a weight-based dose of contrast. The last limitation is the variable CM administration site in the leg: the information about the exact location (i.e., inguinal area, calf, dorsal foot, etc) was not available at the time of retrospective data collection. The degree of PA enhancement may be influenced by the length of its precardiac venous course, and a long course may result in the dilution of CM and termination of CM administration before the CT scanning. However, by checking the CT scanning performed in the middle of the infusion of CM or saline chaser, and confirming the presence of nonmixed CM in the IVC in all cases of group A, we eliminated the possibility of poor PA enhancement due to the termination of CM injection before the CT scan. Furthermore, the observed RV attenuation was influenced by the IVC area at narrowest point, which may explain the poor PA enhancement despite continuing CM flow in the precardiac venous system. Future studies should be designed considering the above limitations.

In conclusion, the rate of inadequate CTPA was higher when CM was administered through the leg than through the arm. Administration through a leg vein resulted in a high false-negative rate in the detection of PE. If administration through a leg vein is unavoidable, other respiratory maneuvers, such as shallow continuous breathing, may improve PA enhancement by promoting IVC flow into the right cardiac chamber, despite the trade-off with decreased lung volume and respiration motion artifacts.
